# Escalator Foundation Bolt Loosening Fault Recognition Based on Empirical Wavelet Transform and Multi-Scale Gray-Gradient Co-Occurrence Matrix

**DOI:** 10.3390/s23156801

**Published:** 2023-07-30

**Authors:** Xuezhuang E, Wenbo Wang

**Affiliations:** Hubei Province Key Laboratory of System Science in Metallurgical Process, Wuhan University of Science and Technology, Wuhan 430081, China; exuezhuang@wust.edu.cn

**Keywords:** empirical wavelet decomposition, bispectrum analysis, fault identification, GGCM, escalator

## Abstract

An escalator is an essential large-scale public transport equipment; once it fails, this inevitably affects the operation of the escalator and even leads to safety concerns, or perhaps accidents. As an important structural part of the escalator, the foundation of the main engine can cause the operation of the escalator to become abnormal when its fixing bolts become loose. Aiming to reduce the difficulty of extracting the fault features of the footing bolt when it loosens, a fault feature extraction method is proposed in this paper based on empirical wavelet transform (EWT) and the gray-gradient co-occurrence matrix (GGCM). Firstly, the Teager energy operator and multi-scale peak determination are used to improve the spectral partitioning ability of EWT, and the improved EWT is used to decompose the original foundation vibration signal into a series of empirical mode functions (EMFs). Then, the gray-gradient co-occurrence matrix of each EMF is constructed, and six texture features of the gray-gradient co-occurrence matrix are calculated as the fault feature vectors of this EMF. Finally, the fault features of all EMFs are fused, and the degree of the loosening of the escalator foundation bolt is identified using the fused multi-scale feature vector and BiLSTM. The experimental results show that the proposed method based on EWT and GGCM feature extraction can diagnose the loosening degree of foundation bolts more effectively and has a certain engineering application value.

## 1. Introduction

Escalators have become an important transportation tool in modern city life. Escalators are commonly used in large shopping malls, railway stations and subway stations, and other large buildings, transporting a large number of people and equipment every day. And, the safety of escalators is closely related to the safety of the public. Once an escalator malfunction occurs, it may affect the operation of the escalator to malfunction, in mild cases, or cause serious accidents, which can cause great damage to people’s lives as well as the economy [1,2]. Due to frequent overtime and overload operation, loosening of the anchor bolts of the escalator’s mainframe footing occurs from time to time. As the key part of the escalator, the loosening of the fixing bolts of the mainframe feet leads to periodic shocks when the escalator is operating, which causes the vibration of the escalator coupling system to intensify, in turn affecting the stability of the escalator’s operation, thus endangering the operational safety of the escalator in serious cases [3]. Therefore, the condition monitoring and fault diagnosis of escalator bracket fixing bolts is essential. The vibration signals of the footing of the escalator are usually nonlinear and non-stationary [4,5]. Moreover, due to the complex operating environment, the collected vibration signals often contain a large amount of noise and interference signals, which makes it difficult to effectively extract the features of the early footing bolt loosening fault. How to effectively suppress noise interference and extract effective bolt loosening fault features from nonlinear and non-stationary footing vibration signals is a key problem that must be solved in escalator condition monitoring.

In view of the nonlinear and non-stationary nature of mechanical fault signals and the characteristics of weak early fault characteristics and susceptibility to noise interference, numerous vibration signal fault diagnosis methods have been proposed, such as short-time Fourier transform (STFT), wavelet transform (WT), empirical mode decomposition (EMD), variational mode decomposition (VMD), etc. However, the window function of STFT cannot be adaptively adjusted according to the frequency of the signal itself, which affects the accuracy of fault diagnosis [6,7]. The selection of wavelet basis functions and decomposition layers of WT lacks adaptivity [8,9]. Empirical mode decomposition (EMD) [10] has made a significant breakthrough in vibration signal fault information extraction, but EMD suffers from serious mode aliasing phenomena and endpoint effects and lacks the necessary theoretical foundation [11,12]. In order to surmount the shortcomings of EMD methods, many improved EMD algorithms have been proposed, such as local mean decomposition (LMD) [13], local characteristic-scale decomposition (LCD) [14], ensemble EMD (EEMD) [15], etc. However, these methods cannot completely solve the limitations of EMD. Variational mode decomposition (VMD) [16] overcomes the deficiencies of EMD and LMD. Signal decomposition is transformed into a variational problem to determine the center frequency and bandwidth of the component signals by seeking the optimal solution of the variational problem so as to achieve the effective separation of the component signals. VMD has a sound theoretical basis and can better suppress modal aliasing. However, the combination of parameters and the number of decompositions of the penalty factor need to be determined before decomposition because different combinations of parameters and the number of decompositions can affect the decomposition accuracy of the signal, which poses great difficulties to the accurate decomposition of the signal [17,18].

Recently, Gilles [19] proposed empirical wavelet transform (EWT) to perform the adaptive decomposition of non-stationary signals. The main idea is to extract each mode component of the signal by constructing a series of suitable band-pass filter banks.

Compared with EMD and VMD methods, EWT has a reliable mathematical theory foundation, and the filter frequency band is self-adaptive according to the signal spectrum, which avoids mode aliasing, and the end effect is better. At the same time, because EWT is not decomposed in an iterative way, the decomposition speed is very fast. Due to the above advantages, EWT has been widely used in the identification of the fault information of rolling bearings [20,21] and fan bearings [22]. In this paper, EWT is introduced into the processing of the foundation vibration signals and is used to perform the multi-scale decomposition of foundation vibration signals. In order to better suppress the influence of noise and non-correlated vibration on EWT band division, the EWT is improved by using the Teager energy operator and multi-scale peak location so that it can separate different modes of the bolt loosen fault signal more effectively.

After decomposing the machine feet vibration signal into a group empirical mode function (EMF) by EWT, how to accurately extract the fault features from each mode component is another key issue in the identification of bolt loosening fault. The EMFs decomposed by EWT contain rich feature information, and different features represent different physical meanings. Choosing a suitable feature extraction method can significantly improve the recognition accuracy. The gray-gradient co-occurrence matrix (GGCM), based on bispectrum analysis, is a very effective fault feature extraction method. The gray-gradient co-occurrence matrix contains all the phase and texture information of processed signals and can better suppress the influence of Gaussian noise [22,23]. Liu et al. [24] applied the GGCM to the detection of microcracks under mixed frequency excitation. Wang et al. [25] used the fractional GGCM of bispectrum analysis to identify fault characteristics, and the results showed that the GGCM can effectively extract the fault features from small cracks. Xu et al. [26] applied the GGCM based on the bispectrum analysis method to identify and analyze the signals of bearing; the experimental results in this study verify the effectiveness of the GGCM based on the bispectrum method in microcrack feature extraction.

Based on the above analysis, a feature extraction method for escalator foundation bolt loosening faults based on EWT and the gray-gradient co-occurrence matrix is proposed in this paper; the method ensures the identification of foundation bolt loosening faults and determines their degree of looseness. Firstly, in order to reduce the influence of noise and irrelevant components, the Teager energy operator and multi-scale peak location method are used to determine the EWT spectral segmentation boundary, and the improved EWT is used to decompose the foundation vibration signal into a group of empirical mode functions (EMFs). Then, in order to avoid complex parameter settings and accurately extract the fault features of loose foundation bolts, the bispectrum and grayscale gradient co-occurrence matrix of each EMF are calculated, and six fault features are extracted through the GGCM of each EMF. Finally, the fault features of all EMFs are fused into an 18-dimensional fault feature vector, and bidirectional long short-term memory (BiLSTM) is used to identify the loosening status of foundation bolts. Experimental analysis is conducted using the measured vibration signals of the escalator foundation, and the results show that the proposed method can effectively identify the looseness faults of the foundation bolts and determine the degree of bolt looseness.

The remainder is structured as follows. Section 2 introduces the basic theory of EWT and Bi-LSTM. The fault features of foundation bolt loosening are extracted using the GGCM based on bispectrum in Section 3. Section 4 constructs the bolt loosening diagnosis model based on EWT and the multi-scale GGCM. An experiment verification and performance comparisons are shown to demonstrate the effectiveness and validity of the proposed method in Section 5. Finally, a conclusion is drawn in Section 6.

## 2. Theories of EWT and BI-LSTM

### 2.1. EWT and Spectrum Division

#### 2.1.1. EWT

Empirical wavelet transform (EWT) is constructed on the basis of wavelet theory. EWT consists of two important steps: First, the adaptive partitioning of the signal spectrum; then, the signal is decomposed using an orthogonal wavelet filter bank to obtain a modal component signal with tight support characteristics. It is assumed that the spectrum range of the vibration signal after Fourier transform is [0,π]. By dividing the whole spectrum into N segments, each segment of the spectrum is denoted as $\Lambda_{n}=[\omega_{n-1},\omega_{n}]$ n=1,2,…,N. $\omega_{0}$ and $\omega_{N}$ are the left and right boundaries of the spectrum division, respectively. Then, the whole spectrum of the signal can be represented as $\cup_{n=1}^{N}\Lambda_{n}=[0,\pi ]$. EWT is a band-pass filter defined on each spectrum $\Lambda_{n}$. According to the theory of the wavelet, the scaling function φ(x) and wavelet function ψ(x) of EWT are defined in the frequency domain as follows [19]:

(1) $${\hat{\varphi }}_{n}(\omega )=\left\{\begin{array}{c} 1;\left|\omega \right|\leq (1-\lambda )\omega_{n}\\ \begin{array}{l} \cos\left[\frac{\pi }{2}\beta \left(\frac{1}{2\lambda \omega_{n}}(|\omega |-(1-\lambda )\omega_{n})\right)\right]\\ (1-\lambda )\omega_{n}\leq \left|\omega \right|\leq (1+\lambda )\omega_{n} \end{array}\\ 0;others \end{array};\right.$$

(2) $${\hat{\psi }}_{n}(\omega )=\left\{\begin{array}{c} 1;(1+\lambda )\omega_{n}\leq \left|\omega \right|\leq (1-\lambda )\omega_{n+1}\\ \begin{array}{l} \cos\left[\frac{\pi }{2}\beta \left(\frac{1}{2\lambda \omega_{n+1}}(|\omega |-(1-\lambda )\omega_{n+1})\right)\right];\\ (1-\lambda )\omega_{n+1}\leq \left|\omega \right|\leq (1+\lambda )\omega_{n+1} \end{array}\\ \begin{array}{l} \sin\left[\frac{\pi }{2}\beta \left(\frac{1}{2\lambda \omega_{n}}(|\omega |-(1-\lambda )\omega_{n})\right)\right]\\ (1-\lambda )\omega_{n}\leq \left|\omega \right|\leq (1+\lambda )\omega_{n+1} \end{array}\\ 0;others \end{array}\right.$$

where $\beta_{n}=y^{4}(35-84y+70y^{2}-20y^{3})$, 0<λ<1, and $\lambda <\underset{n}{\min}\left(\frac{\omega_{n+1}-\omega_{n}}{\omega_{n+1}+\omega_{n}}\right)$.

The decomposition of EWT is similar to that of classical wavelets. The decomposed detail and approximation coefficients can be defined as:

(3) $$\begin{array}{l} W_{g}^{\varepsilon }(n,x)=<g(x),\psi_{n}(x)>\\ =\int g(\tau )\psi_{n}(\tau -x)d\tau =F^{-1}[\hat{g}(\omega ){\hat{\psi }}_{n}(\omega )] \end{array}$$

(4) $$\begin{array}{l} W_{g}^{\varepsilon }(0,x)=<g(x),\phi_{1}(x)>\\ =\int g(\tau )\phi_{1}(\tau -x)d\tau =F^{-1}[\hat{g}(\omega ){\hat{\phi }}_{1}(\omega )] \end{array}$$

where $F^{-1}(\cdot )$ denotes the inverse Fourier transform, and ${\hat{\phi }}_{1}(\omega )$ and ${\hat{\psi }}_{n}(\omega )$ are obtained by Equations (1) and (2). In empirical wavelet transform, the reconstructing formula is:

(5) $$\begin{array}{l} g(x)=W_{g}^{\varepsilon }(0,x)\phi_{1}(x)+\sum_{n=1}^{N}W_{g}^{\varepsilon }(n,x)\psi_{n}(x)\\ =F^{-1}[{\hat{W}}_{g}^{\varepsilon }(0,\omega ){\hat{\phi }}_{1}(\omega )+\sum_{n=1}^{N}{\hat{W}}_{f}^{\varepsilon }(n,\omega ){\hat{\psi }}_{n}(\omega )] \end{array}$$

According to Formulas (3) and (4), the empirical mode function (EMF) formula after EWT decomposition can be calculated using the following formulas:

(6) $$\left\{\begin{array}{c} g_{0}(x)=W_{g}^{\varepsilon }(0,x)\phi_{1}(t)\\ g_{n}(x)=W_{g}^{\varepsilon }(n,x)\psi_{n}(x) \end{array}\begin{array}{c} n=1,2\ldots ,N \end{array}\right.$$

A series of empirical mode functions (EMFs) can be obtained after the machine feet vibration signal is decomposed using EWT. Moreover, bispectrum analysis is performed for each EMF to extract the fault eigenvectors for each mode.

#### 2.1.2. Spectrum Division Improvement in EWT

According to the basic concept of EWT, whether the component signals obtained by EWT are single or not largely depends on the adaptive division of the Fourier spectrum. If the boundary is detected by the basic “locmaxmin” method in EWT, the detection boundary is the minimum value between the two maximum values. Therefore, if the signal is severely affected by noise interference, the “locmaxmin” method can easily cause some frequency bands to be too finely divided while others are not able to be reasonably divided. For the fault signal of escalator foot looseness, the sideband of fault vibration will be easily divided into different kinds of components, thereby resulting in unreasonable spectrum segmentation and the unfavorable demodulation analysis of fault features. In this paper, in order to better separate the fault signals of different modes, the Teager energy operator is used to concentrate the energy for the Fourier spectrum. Meanwhile, the abovementioned method can reduce the influence of noise and irrelevant components. Multi-scale peak search and location is utilized to identify the spectrum segmentation boundary of EWT adaptively while the mode decomposition ability is enhanced. The concrete steps based on the Teager energy operator and multi-scale peak location (TMPL-EWT) are as follows:

Step 1: Utilize Teager energy operator to concentrate energy on the spectrum.

For discrete-time signals x(n), the Teager energy operator is defined as [27]:

(7) $$\Psi (x(n))={\left[x(n)\right]}^{2}-x(n-1)x(n+1)$$

Let the Fourier transform amplitude of the footing vibration signal be $\hat{f}(n)$. Then, the Teager energy operator transformation result is:

(8) $$\Psi (\hat{f}(n))={\left[\hat{f}(n)\right]}^{2}-\hat{f}(n-1)\hat{f}(n+1)$$

It is more reliable to determine the spectrum segmentation boundary from the perspective of spectrum energy than solely from the perspective of amplitude; at the same time, the interference of noise on segmentation is greatly decreased.

Step 2: Multi-scale peak localization

In order to accurately locate the position of the “peak” in the energy spectrum $\Psi (\hat{f}(n))$, this paper proposes a multi-scale peak search and location method whose basic idea is to use a set of window functions with different widths to smooth the data. Because the local maximum on different scales is related, the accurate identification of the peak value can be conducted by the relevance.

For a given N×1 dimensional input sequence s, the peak discriminant criterion is defined as Ω, which is a N×1 dimension vector and its initialization is $\Omega ={\left\{0\right\}}_{N\times 1}$.

(i) Detect all local maximum points of s, form a set Γ, and make the first update to the discriminant criterion Ω, so that: s(k)→Ω(k), ∀k∈Γ.
(ii) Use M window functions $W\left(L_{m}\right)$ with different widths to smooth the signal s and obtain the corresponding smoothing results $s_{m}$:
  $$s_{m}=W(L_{m})*s$$
  where ∗ denotes the convolution operation, and W(·) denotes the standardized Gaussian window function. $L_{m}=\frac{mN}{400}(m=1,2,\ldots ,M)$ denotes the width of the m-th window function, and N denotes the length of the vibration signal.
(iii) After each smoothing, the local maximum is redetected to obtain a new set $\Gamma_{m}$. Combine the new set of local maximum $\Gamma_{m}$ with the initial set Γ of local maximum, and increase the value of the corresponding position in the discrimination criteria Ω:
  (9) $$\Omega (k)=\Omega (k)+s(k)\cdot m,\forall k\in \Gamma_{m}$$
(iv) After m scale iterations, the position of the spectral “peak” can be determined by Ω. For a given number P of peak detections, the first P points with the highest values are taken in Ω.

Step 3: EWT spectrum division

The midpoint of the selected adjacent peak positions is used as the empirical wavelet spectrum segmentation boundary. Corresponding empirical wavelet filter banks are established, and corresponding modal components can be extracted.

**Figure 1:**
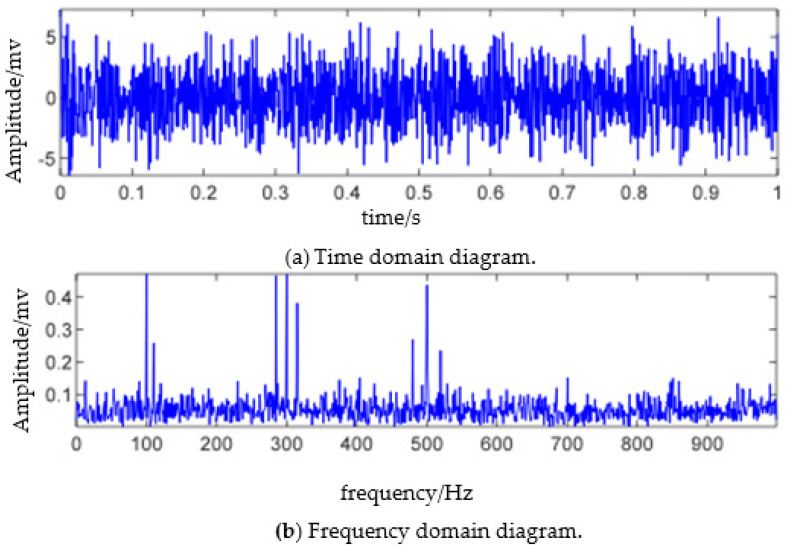
Simulated signal and FFT spectrum.

To verify the effectiveness of this method, a set of simulation fault signals are established for experiment analysis. The simulation signal is as follows:

(10) $$\left\{\begin{array}{c} s_{1}(t)=(1+0.7\sin(20\pi t)\cos(200\pi t+0.7\sin(20\pi t))\\ s_{2}(t)=(0.9+1.8\sin(30\pi t)\sin(600\pi t+0.5\cos(30\pi t))\\ s_{3}(t)=(1+\sin(40\pi t))\cos(1000\pi t+0.8\sin(40\pi t))\\ s(t)=s_{1}(t)+s_{2}(t)+s_{3}(t)+n(t) \end{array}\right.$$

where n(t) denotes white noises with zero mean value. The simulation signal s(t) consists of three mode signals whose center frequencies are 100 Hz, 300 Hz, and 500 Hz, respectively. After adding in zero-mean Gaussian white noise with a signal-to-noise ratio of dB to the simulation signal, the time domain and frequency domain information of the simulation signal are presented in Figure 1, where the sampling frequency is set as $f_{s}$ = 2000 Hz, and the sampling time is set as 1 s.

**Figure 2:**
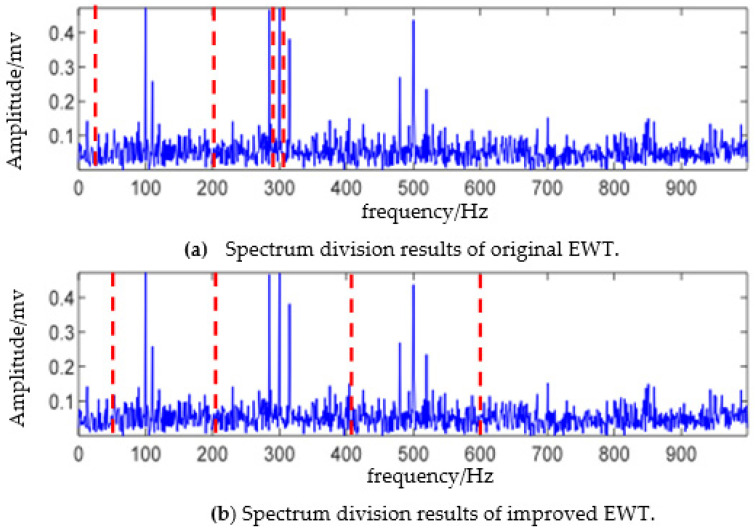
Comparison of spectrum division results of EWT.

The number of components of the simulation signal is three. Considering the influence of noise, the modal component estimator is set as N=4 here. The results of directly using the EWT algorithm to perform the boundary detection and spectrum segmentation on the simulation signal are presented in Figure 2a. Due to the large spectral amplitude of component two, when using “Locmaxmin” for boundary detection, the detected boundaries are all concentrated in component two with the phenomenon of over decomposition. Moreover, part of the modulation signal from component three and component two is mixed in the same mode. Therefore, that is not conducive to the demodulation analysis. The results of using the TMPL-EWT algorithm to perform boundary detection and spectrum segmentation on the simulation signal are shown in Figure 2b. It can be seen that the TMPL-EWT method effectively detects the resonance frequency bands excited by three intrinsic frequencies, thereby avoiding frequency band breakage caused by the improper segmentation of the original EWT, resulting in the achievement of a better separation of fault information.

### 2.2. BI-LSTM

#### 2.2.1. LSTM

**Figure 3:**
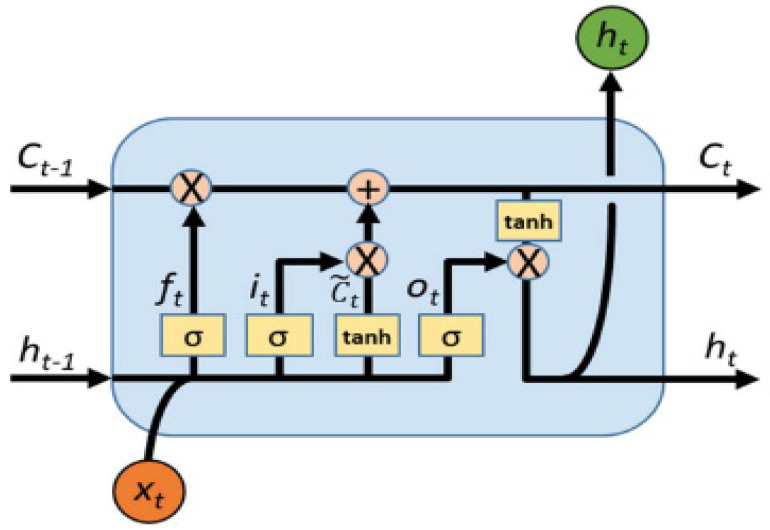
LSTM network structure diagram.

Long short-term memory (LSTM) is an improved model based on the recurrent neural network (RNN). The structure of LSTM is shown in Figure 3, which contains four main gate structures: input gate i, forget gate f, control gate c, and output gate o.

The input gate i determines which new information will be stored in the new cell state, and the calculation of the input gate at moment t is defined as:

(11) $$i_{t}=\sigma (W_{i}\cdot [h_{t-1},x_{t}]+b_{i})$$

The forget gate f determines which information should be ignored from prior memory, and the forgetting gate at moment t is calculated and as defined as:

(12) $$f_{t}=\sigma (W_{f}\cdot [h_{t-1},x_{t}]+b_{f})$$

The control gate updates when the control unit status changes from $c_{t-1}$ to $c_{t}$ according to Equations (11) and (12).

(13) $${\tilde{c}}_{t}=\tanh(W_{c}•[h_{t-1},x_{t}]+b_{c})$$

(14) $$c_{t}=f_{t}\circ c_{t-1}+i_{t}\circ {\tilde{c}}_{t}$$

The output gate o generates the output and updates hidden vector $h_{t-1}$. The control process of the output gate is defined as:

(15) $$o_{t}=\sigma (W_{o}•[h_{t-1},x_{t}]+b_{o})$$

(16) $$h_{t}=o_{t}\circ \tanh(c_{t})$$

In Equations (11)–(16), $h_{t}$ is the final output of the network; ${\tilde{c}}_{t}$ is the current input cell state; $c_{t}$ is the cell state at the current moment; $W_{i}$, $W_{f}$, $W_{c}$, and $W_{o}$ are the weight matrices of the four gated states, respectively; $b_{i}$, $b_{f}$, $b_{c}$, and $b_{o}$ are the bias of each gated state, respectively; σ(·) and tanh(·) are the transfer functions; • represents the inner vector product; and the symbol ∘ denotes multiplication by elements.

#### 2.2.2. BI-LSTM

**Figure 4:**
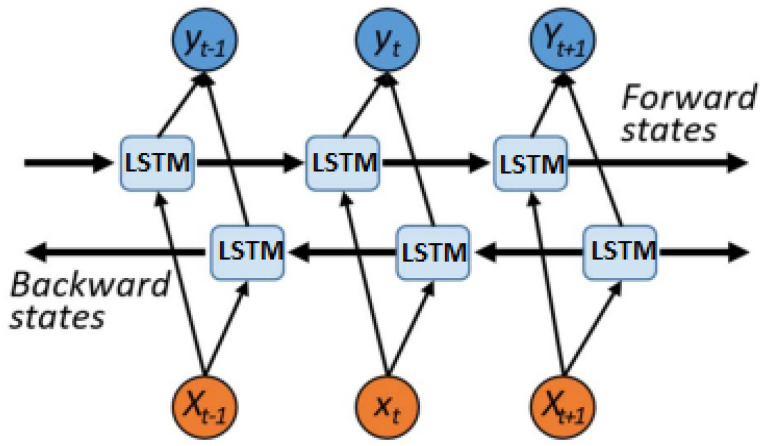
BI-LSTM network structure diagram.

Bidirectional recurrent neural networks (BRNNs) form a bidirectional network structure by adding a backpropagation layer to a recurrent neural network in order to use contextual information simultaneously. In the bidirectional network structure, the RNN units are replaced by LSTM units to form a bidirectional LSTM (Bi-LSTM). The structure of the Bi-LSTM network is given in Figure 4, in which two independent LSTM networks are included to propagate information forwards and backwards, respectively. The Bi-LSTM network has the advantages of both the RNN and LSTM network. Bi-LSTM overcomes the decline problem which the RNN had. Through a parallel forward propagation network and backward propagation network, an estimate of the impact of forward and backward events on current events can effectively be made.

## 3. Multi-Scale Fault Feature Extraction Based on GGCM

### 3.1. Bispectrum Analysis Theory

Bispectrum can effectively suppress Gaussian noise with a high resolution and can obtain the signal amplitude, phase, energy, and other related information. Bispectrum analysis is simple to calculate, but it still contains all the feature information of the higher-order spectrum. Therefore, in this paper, bispectrum analysis is used to extract information about the fault characteristics in the machine feet vibration signal. The steps of bispectrum calculation using the direct method are as follows [23,24]:

(1) The vibration signal to be analyzed is divided into K segments, with each segment containing M samples. So, the signal after segmentation is:
  (17) $$x^{\left(k\right)}(n)=\{x^{\left(k\right)}(0),x^{\left(k\right)}(1),\ldots ,x^{\left(k\right)}(M-1)\},k=1,2\ldots ,K$$
(2) For the kth segment of data $x^{\left(k\right)}(n)$, calculate its discrete Fourier transform.
  (18) $$X^{\left(k\right)}(\lambda )=\frac{1}{M}\sum_{n=0}^{M-1}x^{\left(k\right)}(n)\exp(-j\frac{2\pi n\lambda }{M}),\lambda =0,1\ldots ,M/2;k=1,2\ldots ,K$$
(3) Calculate the third-order autocorrelation coefficients of DFT.
  (19) $$\begin{array}{l} b_{k}(\lambda_{1},\lambda_{2})=\frac{1}{\Delta_{0}^{2}}\sum_{i_{1}=-L_{1}}^{L_{1}}\sum_{i_{2}=-L_{1}}^{L_{1}}X^{\left(k\right)}(\lambda_{1}+i_{1})\cdot \\ \cdot X^{\left(k\right)}(\lambda_{2}+i_{2})\cdot X^{\left(k\right)}(-\lambda_{1}-\lambda_{2}-i_{1}-i_{2}) \end{array}$$
  where $\Delta_{0}=f_{s}/N_{0}$, $M=(2L_{1}+1)N_{0}$, and $f_{s}$ denotes the sampling frequency.
(4) Calculate the bispectrum estimation of the vibration signal.
  (20) $$B(\omega_{1},\omega_{2})=\frac{1}{K}\sum_{k=1}^{K}b_{k}(\omega_{1},\omega_{2})$$
  where $\omega_{1}=\frac{2\pi f_{s}}{N_{0}}\lambda_{1}$ and $\omega_{2}=\frac{2\pi f_{s}}{N_{0}}\lambda_{2}$.

It can be seen from the definition that the bispectrum is a complex spectrum with two frequency variables, $\omega_{1}$ and $\omega_{2}$. Bispectrum has 12 symmetrical regions in the frequency plane composed of $\omega_{1}$ and $\omega_{2}$. Only the bispectrum values in the main region need to be calculated, and then, all the bispectrum values in the ($\omega_{1}$, $\omega_{2}$) plane can be calculated according to its symmetry.

### 3.2. Bispectrum Analysis of EWT for Escalator Foundation Vibration Signal

With regard to the complexity of the environment, the actual vibration signal collected from the foundation of the escalator usually contains interfering noise. Bispectrum analysis can only effectively suppress Gaussian noise, but it is powerless against non-Gaussian noise. Therefore, EWT is used to remove the effect of non-Gaussian noise from the signal before performing bispectrum analysis. After the decomposition of the machine feet vibration signal using EWT, a series of EMFs are obtained. Because of the high noise frequency, the noise-containing signal is often concentrated in the highest frequency modal component EMF1 after EWT decomposition [20,21], while the fault feature information is contained in the remaining low-frequency modal components. So, this paper discards the first layer of empirical mode function EMF1 and only performs bispectrum analysis on the remaining mode functions to extract the fault feature information hidden in the mode components.

**Figure 5:**
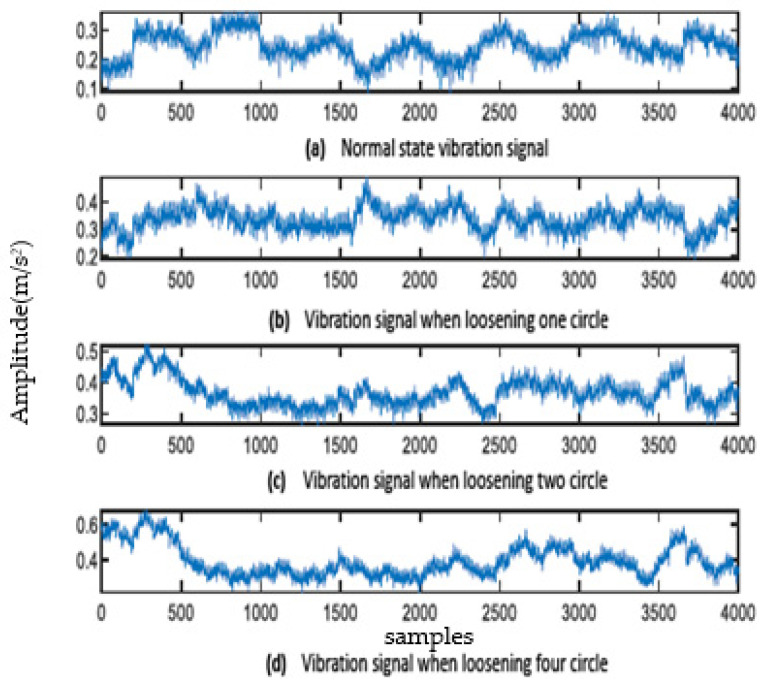
Original machine feet vibration signal.

**Figure 6:**
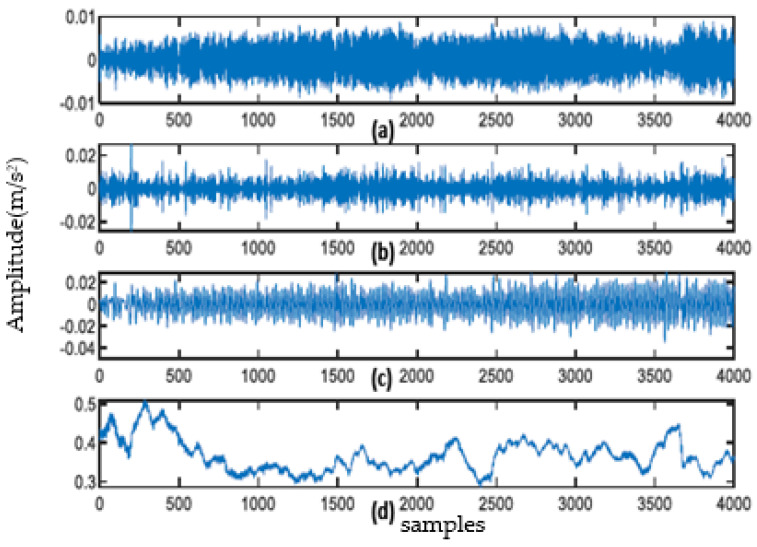
Results of EWT decomposition. (**a**) ${\mathrm{EMF}}_{1}$; (**b**) ${\mathrm{EMF}}_{2}$; (**c**) ${\mathrm{EMF}}_{3}$; (**d**) ${\mathrm{EMF}}_{4}$.

The sampling frequency of the vibration signal of the escalator base foot is 2000 Hz. The signals of normal, loosening one lap, loosening two laps, and loosening three laps of fixed bolts are collected, respectively, and some of the collected signals are intercepted as shown in Figure 5. EWT decomposition is carried out for each of the four signals, and the number of frequency band intervals for EWT decomposition is taken as four. The decomposed EMFs of the machine feet vibration signal with loosening one lap are shown in Figure 6 (the EMFs of the vibration signal of other cases are similar to this, but due to space limitations, they are not shown one by one).

**Figure 7:**
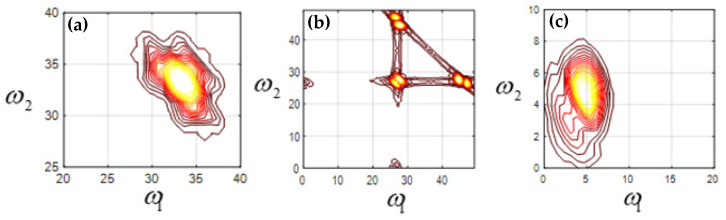
Contour of normal (**a**) ${\mathrm{EMF}}_{2}$; (**b**) ${\mathrm{EMF}}_{3}$; (**c**) ${\mathrm{EMF}}_{4}$.

**Figure 8:**
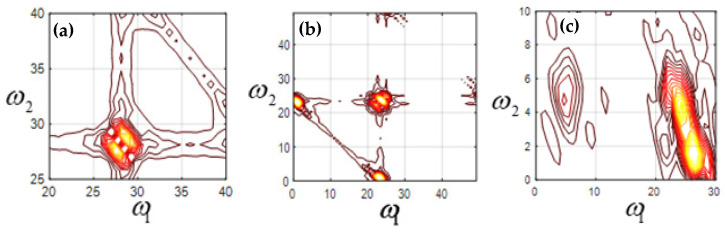
Contour of loosening 1 lap: (**a**) ${\mathrm{EMF}}_{2}$; (**b**) ${\mathrm{EMF}}_{3}$; (**c**) ${\mathrm{EMF}}_{4}$.

**Figure 9:**
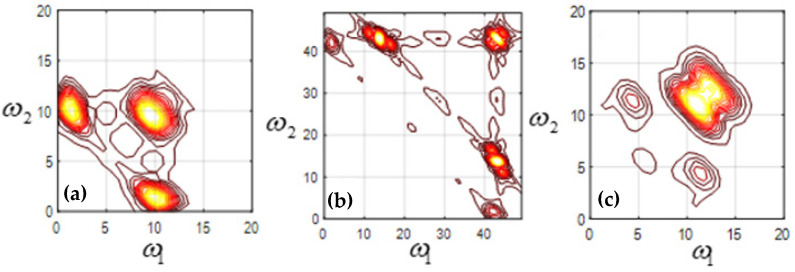
Contour of loosening 2 laps (**a**) ${\mathrm{EMF}}_{2}$; (**b**) ${\mathrm{EMF}}_{3}$; (**c**) ${\mathrm{EMF}}_{4}$.

**Figure 10:**
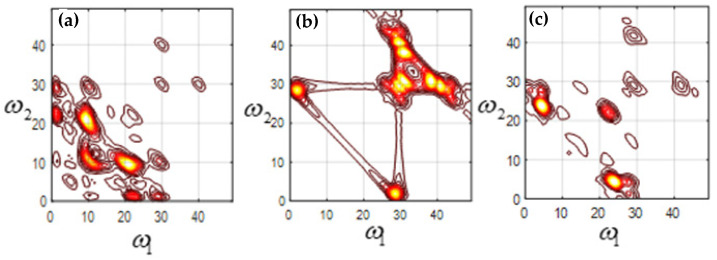
Contour of loosening 3 laps (**a**) ${\mathrm{EMF}}_{2}$; (**b**) ${\mathrm{EMF}}_{3}$; (**c**) ${\mathrm{EMF}}_{4}$.

The bispectrum analysis of mode function 2 (EMF2), mode function 3 (EMF3), and mode function 4 (EMF4) of four kinds of signals is carried out, respectively. Figure 7 shows the two-dimensional contour plot of bispectrum analysis of the machine feet vibration signal when the fixed bolt is normal. Figure 8, Figure 9 and Figure 10 show the two-dimensional contour plot of bispectrum analysis of the footing vibration signal when the fixed bolt is loosening for one, two, and three laps, respectively. From Figure 7, Figure 8, Figure 9 and Figure 10, it can be seen that the two-dimensional spectrum of the escalator footing bolt shows obvious differences under normal working conditions and loose operating conditions. Compared with EMF2 and EMF3, EMF4 contains the least noise and interference signals, and EMF4 also concentrates the majority of the energy of the foundation vibration. Observing the bispectrum of EMF4 (Figure 5c, Figure 6c, Figure 7c and Figure 8c), it can be seen that when the foundation bolt is normal, the energy of the bispectrum of the foundation vibration signal converges towards the center. As the bolts gradually loosen, the energy of the bispectrum of the foundation vibration signal gradually expands outward. As the degree of bolt loosening increases, the frequency extension range of the bispectrum also gradually increases.

### 3.3. Fault Feature Extraction Based on Gray-Gradient Co-Occurrence Matrix

In order to automatically identify whether the bolt is loose and confirm the degree of loosening, the feature information contained in the bispectrum coefficients needs to be extracted. The two-dimensional contour plot of the bispectrum analysis contains the basic feature information of the vibration signal, while its gradient plot depicts the edge and abrupt change information of the contour map. If both the two-dimensional contour plots and gradient plots are combined for feature extraction, more accurate fault characteristics can be obtained. Based on the fact that the grey-gradient co-occurrence matrix (GGCM) [28] can describe both the gray information and the gradient information in the image, this paper constructs a grey-gradient co-occurrence matrix for the two-dimensional contour plot of the bispectrum analysis of vibration signals. The GGCM is used to extract the characteristic parameters of the bispectrum analysis contour map as the fault characteristics when the bolt is loose.

#### 3.3.1. Normalization of Gray Matrix and Gradient Matrix

After the gray processing of the bispectrum analysis two-dimensional contour plot, the gray matrix f(m,n) is obtained. Because the pixel values of the gray matrix are between [0,255], there is no need for normalization. In this section, only the gradient matrix of the two-dimensional contour plot is normalized. Let g(m,n) be the pixel value of point (m,n) in the gradient matrix.

(21) $$g(p,q)=\sqrt{g_{x}^{2}(p,q)+g_{y}^{2}(p,q)}$$

where:

$$\begin{array}{l} g_{x}(p,q)=f(p+1,q-1)+2f(p+1,q)+f(p+1,q+1)\\ -f(p-1,q-1)-2f(p-1,q)-f(p-1,q+1) \end{array}$$

$$\begin{array}{l} g_{y}(p,q)=f(p-1,q+1)+2f(p,q+1)+f(p+1,q+1)\\ -f(p-1,q+1)-2f(p,q-1)-f(p+1,q-1) \end{array}$$

p=1,2,…,P, q=1,2,…,Q, P,Q represent the number of rows and columns of the gray matrix.

Let $g_{\max}$ be the maximum value in the gradient image and $L_{g}$ be the expected maximum gradient value after normalization. Then, the normalized gradient matrix is:

(22) $$G(p,q)=INT[g(p,q)\cdot L_{g}/g_{\max}]+1$$

In this paper, $L_{g}=64$. After normalization, two normalization matrices are obtained: gray normalization matrix F(p,q)=f(p,q) and gradient normalization matrix G(p,q).

#### 3.3.2. Generation of GGCM

The gray-gradient co-occurrence matrix based on bispectrum analysis is C=c(x,y).

Then, its element c(x,y) is defined as the total number of pairs of image points with pixel values x in the normalized grey matrix F(p,q) and pixel values y in the normalized gradient matrix G(p,q). c(x,y) is equal to the number of pairs of image points that make x=F(p,q) and y=G(p,q). In order to facilitate texture feature extraction of the GGCM, it is necessary to normalize it. Let the element value of the normalized GGCM be $\hat{c}(x,y)$, then we have:

(23) $$\hat{c}(x,y)=\frac{c(x,y)}{\sum_{x=0}^{255}\sum_{y=0}^{63}c(x,y)}$$

#### 3.3.3. Texture Feature Extraction with Gray-Gradient Co-Occurrence Matrix

More feature information can be extracted from the GGCM of bispectrum analysis [25].

But if too many feature parameters are selected, this can lead to excessive computational effort and affect the accuracy of the fault identification. Therefore, in this section, six of these feature values are selected as the fault feature vectors for bolt loosening. The equations for each of the features are as follows:

1. Small-gradient dominance:

(24) $$T_{1}=\sum_{x=0}^{255}\sum_{y=0}^{63}\frac{\hat{c}(x,y)}{y^{2}}/\sum_{x=0}^{255}\sum_{y=0}^{63}\hat{c}(x,y)$$

2. Inhomogeneity of the grey distribution:

(25) $$T_{2}=\sum_{x=0}^{255}[\sum_{y=0}^{63}\hat{c}(x,y){]}^{2}/\sum_{x=0}^{255}\sum_{y=0}^{63}\hat{c}(x,y)$$

3. Inhomogeneity of the gradient distribution:

(26) $$T_{3}=\sum_{y=0}^{63}[\sum_{x=0}^{225}\hat{c}(x,y){]}^{2}/\sum_{x=0}^{255}\sum_{y=0}^{63}\hat{c}(x,y)$$

4. Grey entropy:

(27) $$T_{4}=-\{\sum_{x=0}^{255}[\sum_{y=0}^{63}\hat{c}(x,y){]}^{2}\cdot \log[\sum_{y=0}^{63}\hat{c}(x,y)]\}$$

5. Gradient entropy:

(28) $$T_{5}=-\{\sum_{y=0}^{63}[\sum_{x=0}^{255}\hat{c}(x,y){]}^{2}\cdot \log[\sum_{y=0}^{63}\hat{c}(x,y)]\}$$

6. Mixed entropy:

(29) $$T_{6}=\sum_{x=0}^{255}\sum_{y=0}^{63}\hat{c}(x,y)\log[\hat{c}(x,y)]$$

The results of randomly selecting the data from normal state, loosening one lap, loosening two laps, and loosening three laps and calculating the normalized values of EMF2, EMF3, and EMF4 texture features after EWT decomposition are shown in Table 1.

**Figure 11:**
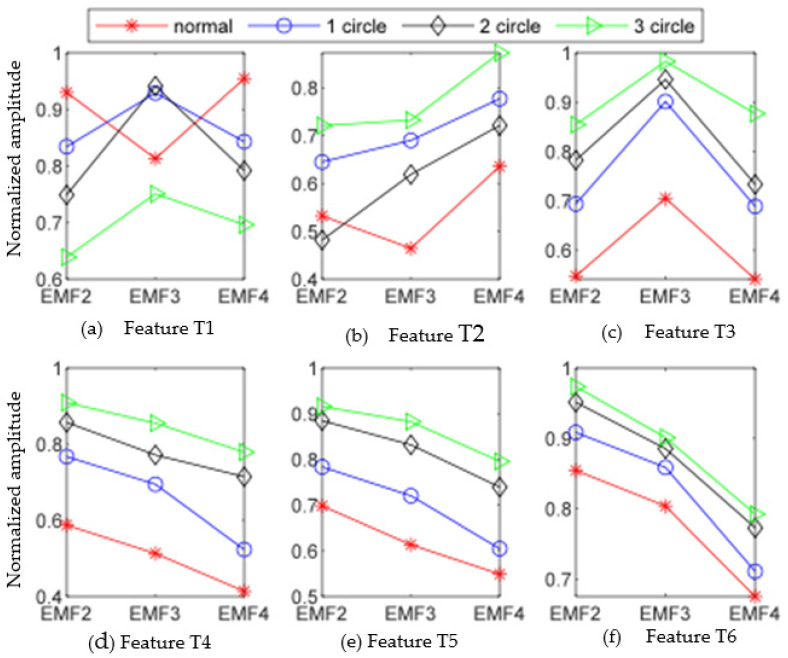
Graphs of six feature vectors based on GGCM.

By analyzing the data in Table 1, it can be found that there are significant differences in the values of texture feature parameters with different degrees of looseness. In order to analyze the experimental results more intuitively, the values of small gradient advantage, non-uniformity of gray distribution, non-uniformity of gradient distribution, gray entropy, gradient entropy, and entropy of mixing are analyzed as a line chart, and the results are shown in Figure 11.

By analyzing Figure 11a, it can be found that there is a better discrimination between the small gradient dominance values of EMF2 and EMF4 components in different states when the foot of the escalator is in a normal, loose state of one turn, loose state of one turns, and loose state of three turns. However, there is a certain degree of overlap between the small gradient advantage values of EMF3 components. It can be seen from Figure 11b that when the foundation bolts are loosened for one circle, two circles, and three circles, respectively, there is a clear distinction between the uneven gray distribution of EMF2, EMF3, and EMF4. For signals in a normal state, the uneven distribution of EMF3 and EMF4 gray-scale is also well differentiated from the fault signal, but there is a certain degree of overlap between the uneven distribution of EMF2 gray-scale in normal state and the fault signal. It can be seen from Figure 11c that when the footing is loosened for one circle, two circles, and three circles, respectively, there is obvious discrimination between the uneven gray distribution of EMF2, EMF3, and EMF4. When the footing is in different states, there is an obvious separation between the gradient distribution nonuniformity feature vectors, and there is no aliasing.

It can also be seen from Figure 11d–f that when the footing is in different states, the eigenvectors composed of gray entropy, gradient entropy, and entropy of mixing of each modal component also have high discrimination, and there is no aliasing between feature vectors. Therefore, combining Table 1 and Figure 11, it can be seen that the six feature values extracted through the gray co-occurrence matrix are effective and can effectively distinguish the fault status of the footing.

After obtaining the six normalized texture feature values of the k-th modal component $EMF_{k}$ (k=2,3,4) of the foundation vibration signal, the texture features ($T_{1}^{EMF_{k}},T_{2}^{EMF_{k}},\ldots ,T_{6}^{EMF_{k}}$) of the three modal components are fused, and the fused 18-dimensional fault feature vector is $T_{1\times 18}$:

(30) $$T_{1\times 18}=[T_{1}^{EMF_{2}},T_{1}^{EMF_{3}},T_{1}^{EMF_{4}},T_{2}^{EMF_{2}},T_{2}^{EMF_{3}},T_{2}^{EMF_{4}},\ldots ,T_{6}^{EMF_{2}},T_{6}^{EMF_{3}},T_{6}^{EMF_{4}}]$$

**Figure 12:**
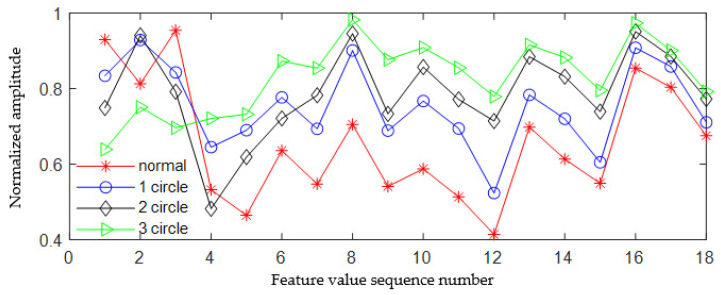
The fused 18-dimensional GGCM feature vector.

The fused multi-scale fault feature vector $T_{1\times 18}$ is used as an input, and a bidirectional long short-term network (Bi LSTM) is used as a classifier to identify different degrees of fundamental motion faults. We randomly select a set of data from normal state, loose one circle, loose two circles, and loose three circles. After EWT decomposition, the texture features of the three modes are fused, and the fused results are shown in Figure 12.

From Figure 12, it can be seen that apart from individual features, the fused feature vectors of different states have a relatively clear degree of differentiation. The fused 18-dimensional feature vectors can effectively diagnose and recognize the loose footings in one, two, three, and normal states.

## 4. Bolt Loosening Fault Diagnosis Model Based on EWT and GGCM

**Figure 13:**
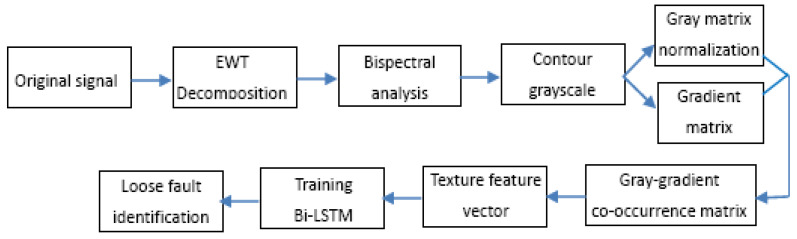
Process diagram of bolt loosening fault diagnosis.

In this section, bidirectional LSTM is used to demonstrate fault identification for the loosening of an anchor bolt of an escalator. Based on the multi-scale GGCM feature and Bi-LSTM, the process of anchor bolt loosening fault diagnosis is shown in Figure 13. The specific steps of bolt loosening fault identification are as follows:

Step 1: EWT is used to decompose each training sample into four levels. And, the last three order empirical mode functions (EMFs) are retained;

Step 2: Bispectrum analysis is performed on the last three order EMFs (EMF_2_, EMF_3_, EMF_4_) of each training sample. The gray matrix and gradient matrix are constructed by the two-dimensional contour plot of bispectrum analysis, and the GGCM is constructed;

Step 3: Six texture features are extracted from the GGCM of EMF_k_ (k = 2, 3, 4) to form the 18-dimensional fault feature vector of training samples.

Step 4: The Bi-LSTM network is trained with training data. And, an anchor bolt loosening fault diagnosis model is established.

## 5. Analysis of Experimental Results

### 5.1. Collection of Experimental Data and Evaluation Index

#### 5.1.1. Experimental Data

In this section, simulation experiments are conducted on a Schindler S9700-30 escalator model with a vibration sensor sampling frequency of 2000 Hz and an acquisition time of 8 s. The vibration sensor is installed on the anchor bolt position of the escalator through the thread.

By adjusting the loosening degree of the anchor bolt, a total of 800 sets of experimental data were collected, including 200 groups of data when the anchor bolt was normal, 200 sets of data when the fixed bolt was loosening one lap, 200 sets of data when the fixed bolt was loosening two laps, and 200 groups of data when the fixed bolt was loosening three laps. According to the escalator product characteristics, the frequency range of the anchor bolt vibration is determined to be between 1 and 5 khz. Therefore, a low-pass filter is used to filter the high-frequency part to avoid the interference signal being introduced into the next level. The filter circuit design uses an active filter circuit to filter out the spurious signal and then amplifies the signal at the same time. During the collection process, a marker line is first drawn on the mainframe anchor bolt not only to record the initial position of the mainframe anchor bolt but also to avoid excessive or inappropriate loosening of the mainframe anchor bolt. After drawing the marker line, the mainframe foot screw is loosening by one, two, and three laps, respectively, against the marker line and confirmed by measurement that the mainframe anchor bolt is loosened in place.

#### 5.1.2. Evaluation Index

To evaluate the performance of the proposed method, accuracy, precision (P), recall (R), and F1-score were selected as the evaluation indices [29,30]. Accuracy represents the ratio of the number of correctly classified samples to the total number of samples, and its calculation formula is:

(31) $$\mathrm{Accuracy}=\frac{\mathrm{TP}+\mathrm{TN}}{\mathrm{TP}+\mathrm{TN}+\mathrm{FN}+\mathrm{FP}}$$

Precision (P) represents the proportion of samples that are truly positive, and its calculation formula is:

(32) $$\mathrm{Precision}=\frac{\mathrm{TP}}{\mathrm{TP}+\mathrm{FP}}$$

Recall rate (R), as one of the important indicators for model performance evaluation, reflects the proportion of all positive samples detected by the model to all true positive samples, and its calculation formula is:

(33) $$\mathrm{Recall}=\frac{\mathrm{TP}}{\mathrm{TP}+\mathrm{TN}}$$

F1-score is a comprehensive evaluation indicator that can balance the precision and recall of the model and better reflects the robustness of the model; its calculation formula is:

(34) $$F1-\mathrm{score}=\frac{2\mathrm{PR}}{P+R}$$

where TP is the number of positive samples predicted by the model as positive, and TN is the number of negative samples predicted by the model as negative; FN represents the number of negative samples that were incorrectly identified as positive samples, while FP represents the number of negative samples that were incorrectly identified as positive samples.

### 5.2. Comparative Analysis of Different Fault Feature Extraction Methods

EWT decomposition was carried out on the experimentally collected vibration data of the anchor bolt under normal and loosening conditions. EMF2, EMF3, and EMF4 were subjected to bispectrum analysis. And, six texture features were extracted for each layer of EMF using a grey-gradient co-generation matrix to form an 18-dimensional fault feature vector, which was input into the Bi-LSTM network to identify the degree of loosening of the anchor bolt. For the vibration signal when the bolt is normal and when the bolt is loose, the corresponding output is “0” for a normal bolt, “1” for loosening 1 lap, “2” for loosening 2 laps, and “3” for loosening 3 laps. In Bi-LSTM, the number of neurons in the input layer, output layer, and hidden layer are 30, 1, and 20, respectively. The internal parameters of the LSTM were trained using Adam’s algorithm with a learning rate of 0.001, a training number of 1000, and a training target of 0.0001. The parameters of the reverse layer network were the same as those of the forward layer. The fault identification accuracy of different methods was analyzed using a 10-fold cross-validation method. In the 10-fold cross-validation, the eigenvector set of the base vibration signal is divided into ten parts on average: nine of them are taken as training data, and one is taken as test data for the classification experiment. Finally, the average of the 10 experimental results is used as an estimate of the accuracy of the method.

**Figure 14:**
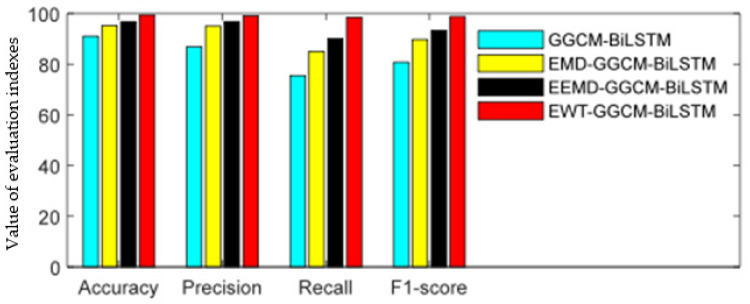
Histogram of the classification performance of four methods.

Figure 14 and Table 2 show the identification accuracy of four methods for the degree of the looseness of foundation bolts when using ten-fold cross validation. It can be seen that the four indexes of the GGCM-BiLSTM method are all the lowest because the direct GGCM method does not perform multi-scale decomposition on the original vibration signal and directly extracts fault features, which affects the accuracy of fault feature extraction. The four evaluation indices of the EMD-GGCM-BiLSTM method are significantly higher than those of the GGCM-BiLSTM method. This is because EMD performs modal separation on the original vibration signal, which to some extent suppresses noise and can more accurately extract fault features. The EEMD-GGCM-BiLSTM method shows a certain improvement in overall recognition performance compared to the EMD-GGCM-BiLSTM method, but this improvement is not significant. The proposed EWT-GGCM-BiLSTM model achieved better results in terms of the four performance indices. Compared with the EEMD-GGCM-BiLSTM method, which showed a better performance, the accuracy of the proposed method improved by about 2.63%, precision improved by about 2 62%, recall increased by approximately 8.50%, and F1 score increased by approximately 5.49%. The experimental results in Figure 14 and Table 2 indicate that the recognition framework based on EWT and multi-scale GGCM feature extraction can effectively identify the degree of looseness of escalator foundation bolts.

**Figure 15:**
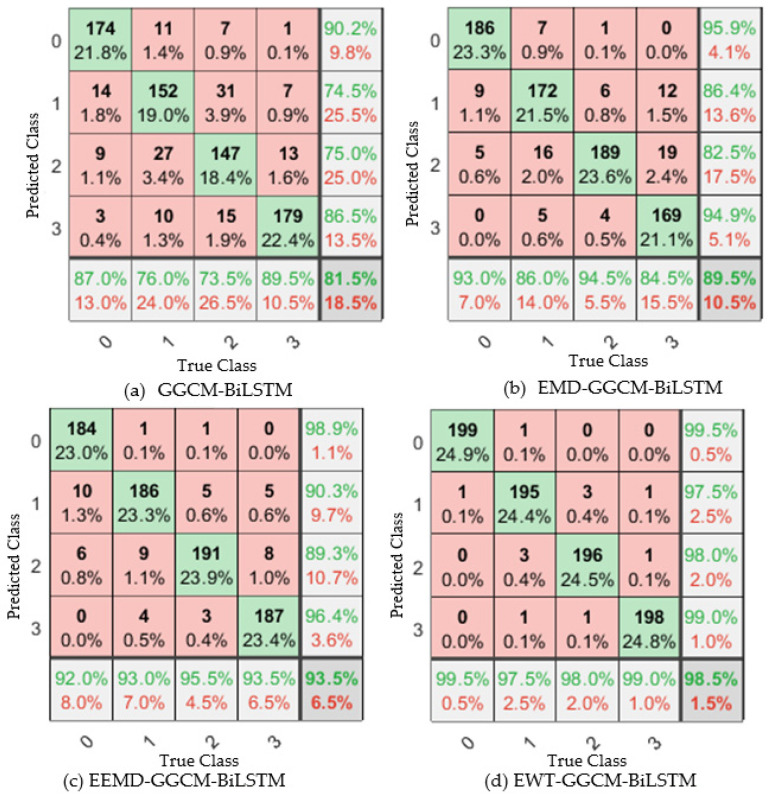
Confusion matrix from ten-fold CV with four methods.

In order to further verify the test results of the proposed model for normal (0), loose 1 circle (1), loose 2 circle (2), and loose 3 circle (3) of the foundation bolt, the confusion matrix shown in Figure 15 was utilized, where the horizontal coordinate represents the real label, and the vertical coordinate represents the prediction result. It can be seen that among all the diagnostic models, the performance of the proposed model in diagnosing bolt loosening faults is far superior to other comparative models, which also verifies the rationality of the proposed model. The reasons for the better identification results of the proposed method are as follows: (1) EWT uses bandpass filter banks to extract each mode component, effectively avoiding mode aliasing, and can separate the fault information of different scales as far as possible. (2) The multi-scale GGCM feature based on EWT can focus on the main vibration features of the loosening of the foundation bolt and suppress the interference of noise and irrelevant background signals on the extracted features. The above experimental results show that high-quality features are the key to improving the identification accuracy of the foundation bolt loosening, and the results of the confusion matrix also verify the rationality of the proposed method.

### 5.3. Comparative with Machine Learning Algorithms

**Figure 16:**
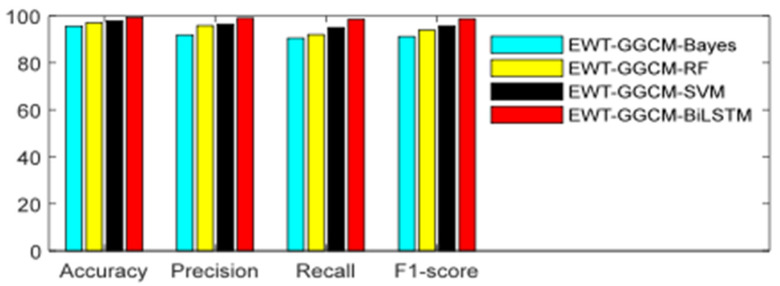
Histogram of the classification performance of four ML methods.

In order to further compare the performance of the proposed method, the proposed method is compared with common machine learning methods, including support vector machine (SVM), random forest (RF), and naive Bayes classifier (NBC). The kernel function of SVM uses the Gaussian radial kernel function, kernel width, and penalty factor determined via grid search. In the random forest model, the values of three important parameters (the number of decision trees, the maximum depth of decision trees, and the minimum sample size that each decision tree can partition) are also selected via grid search. In the naive Bayes classifier model, the prior probability distribution is set to Gaussian distribution. The 10-fold cross validation method is used to analyze the recognition accuracy of different methods, and the average of the ten results is then taken as the final result. The accuracy, precision, recall, and F1-score of the four methods are shown in Figure 16 and Table 3.

**Figure 17:**
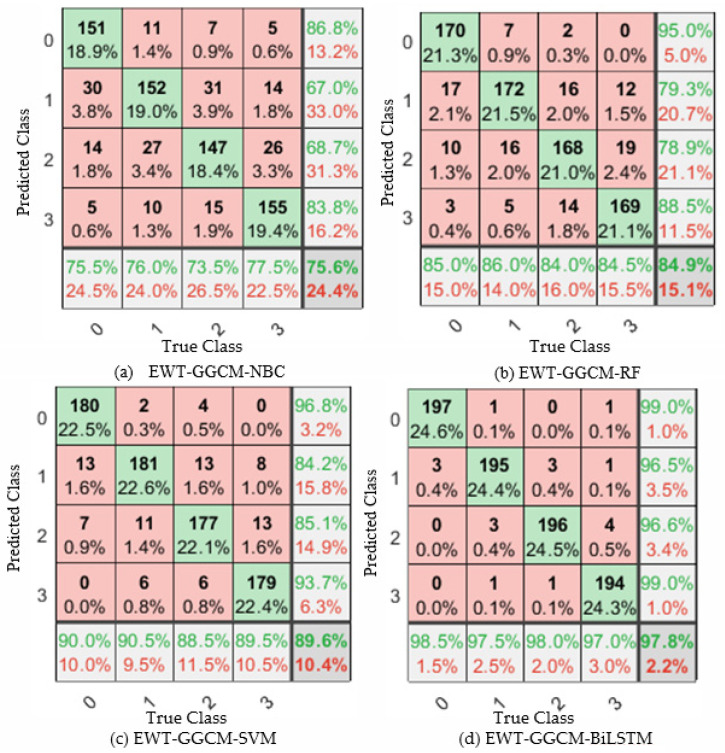
Confusion matrix from ten-fold CV with four ML methods.

From Figure 16 and Table 3, it can be seen that the proposed EMT-GGCM-BiLSTM model has the best recognition performance, with an accuracy of 99.38%, precision of 98.99%, recall of 98.50%, and F1-score of 98.75%. Compared with the SVM method, the proposed method showed an improvement in accuracy of 1.51%, precision of 2.54%, recall of 3.50%, and F1 of 3.03%. Compared with the SVM method, the accuracy, precision, recall, and F1-score of the proposed method improved by 1.51%, 2.54%, 3.50%, and 3.03%, respectively. Compared with the RF method, the accuracy, precision, recall, and F1-score of the proposed method improved by 2.38%, 3.16%, 6.50%, and 4.87%, respectively. Compared with the NBC method, the accuracy, precision, recall, and F1-score of the proposed method improved by 3.75%, 7.11%, 8.00%, and 7.57%, respectively. The above experimental results indicate that the recognition performance of the NBC method is relatively low, and the recognition performance of SVM and RF is basically the same, while the recognition performance of the proposed method is better than that of SVM and RF methods. Therefore, compared with the classical shallow machine learning model, the BiLSTM method based on the deep learning model has certain advantages, with a higher classification accuracy and generalization performance.To further demonstrate the ability of different methods to identify the looseness of foundation bolts, Figure 17 shows the confusion matrix of the identification results of the four methods. As can be seen from Figure 17, it is relatively easy to identify the normal condition and one loosening circle, but the error rate of identifying two loosening circles and three loosening circles is relatively high, which may be due to the similar fault feature of two loosening circles and three loosening circles. Although the proposed method also has the above situation, the overall identification effect is the highest, indicating that the BiLSTM method used can effectively improve the identification ability of different fault feature vectors.

## 6. Conclusions

A diagnostic method based on EWT and multi-scale GGCM feature extraction is proposed for detecting the looseness fault of escalator foundation bolts, and the effectiveness of the proposed method is verified using measured signals.

(1) The Teager energy operator is used to concentrate spectral energy on the spectrum, which greatly reduces the erroneous influence of noise and irrelevant components on spectrum segmentation. The multi-scale “peak” localization method based on the Teager energy operator can detect the segmentation boundary of EWT adaptively, avoid the wrong decision caused by a single “pseudo-value point”, and make the spectrum segmentation more appropriate.
(2) The feature extraction method based on the multi-scale GGCM does not require too many parameter settings, and the feature vector extracted from the multi-scale GGCM can effectively diagnose the loose footing fault. This method is simple, intuitive, and accurate and overcomes the difficulties of fault feature extraction using the traditional method.
(3) The fault recognition method by jointing multi-scale GGCM feature extraction and the BiLSTM classifier has a high recognition accuracy and certain degree of universality, which can be used to identify and warn of foundation bolts loosening faults during the operation of escalators.

In the fault identification of escalator base bolt loosening, the suppression of vibration signal interference noise and the improvement in classifier performance have important effects on the fault identification accuracy. How to use multi-objective optimization algorithm [31] to more effectively suppress interference noise and how to use the attention mechanism to further improve the performance of the BiLSTM classifier are the two research areas that we hope to study in the future.

## Figures and Tables

**Table 1:** Normalized values of the 6 texture features of EMFs.

Texture Features		T_1_	T_2_	T_3_	T_4_	T_5_	T_6_
Normal state	EMF_2_	0.9300	0.5323	0.5475	0.5877	0.6980	0.8543
	EMF_3_	0.8131	0.4646	0.7053	0.5132	0.6135	0.8037
	EMF_4_	0.9544	0.6359	0.5411	0.4140	0.5492	0.6752
Loosing 1 circle	EMF_2_	0.8342	0.6451	0.6937	0.7673	0.7835	0.9084
	EMF_3_	0.9290	0.6900	0.9012	0.6945	0.7202	0.8586
	EMF_4_	0.8431	0.7770	0.6888	0.5236	0.6049	0.7110
Loosing 2 circle	EMF_2_	0.7490	0.4819	0.7825	0.8571	0.8846	0.9513
	EMF_3_	0.9411	0.6189	0.9459	0.7716	0.8318	0.8853
	EMF_4_	0.7919	0.7212	0.7333	0.7143	0.7392	0.7728
Loosing 3 circles	EMF_2_	0.6391	0.7210	0.8546	0.9091	0.9160	0.9736
	EMF_3_	0.7510	0.7323	0.9826	0.8553	0.8826	0.9012
	EMF_4_	0.6964	0.8730	0.8768	0.7791	0.7956	0.7922

**Table 2:** Classification results obtained by four methods.

Models	Accuracy (%)	Precision (%)	Recall (%)	F1 Score (%)
GGCM-BiLSTM	91.00	86.78	75.50	80.75
EMD-GGCM-BiLSTM	95.13	94.97	85.00	89.71
EEMD-GGCM-BiLSTM	96.75	96.37	90.00	93.26
EWT-GGCM-BiLSTM	99.38	98.99	98.50	98.75

**Table 3:** Classification results obtained by four ML methods.

Models	Accuracy (%)	Precision (%)	Recall (%)	F1-Score (%)
EWT-GGCM-NBC	95.63	91.88	90.50	91.18
EWT-GGCM-RF	97.00	95.83	92.00	93.88
EWT-GGCM-SVM	97.87	96.45	95.00	95.72
EWT-GGCM-BiLSTM	99.38	98.99	98.50	98.75

## Data Availability

Not applicable.
